# Supplementary figures and images for: Acute appendicitis manifests as two microbiome state types with oral pathogens influencing severity
Source: Gut Microbes. 2023 Jan 23;15(1):2145845. doi: 10.1080/19490976.2022.2145845 (PMC9879201; doi:10.1080/19490976.2022.2145845)

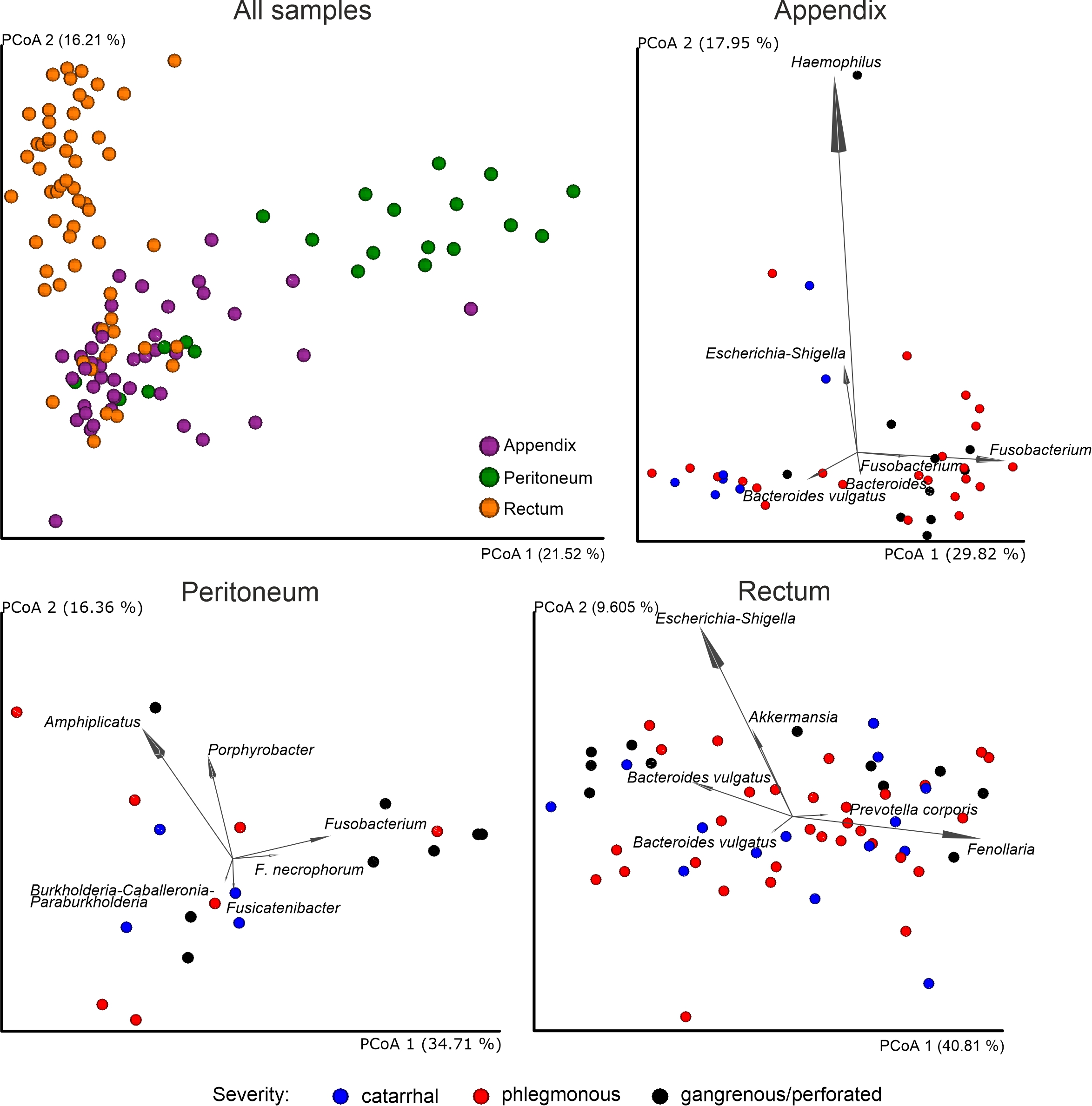

Supplement: Supplemental Material [file KGMI_A_2145845_SM2440.zip › Supplemental Figure 1.jpg]
